# The role of education level on changes in endorsement of medication treatment and perceived public stigma towards psychosis in Hong Kong: comparison of three population-based surveys between 2009 and 2018

**DOI:** 10.1186/s12888-022-04288-1

**Published:** 2022-10-13

**Authors:** Min Yi Sum, Sherry Kit Wa Chan, Yi Nam Suen, Charlton Cheung, Christy Lai Ming Hui, Wing Chung Chang, Edwin Ho Ming Lee, Eric Yu Hai Chen

**Affiliations:** 1 Department of Psychiatry, School of Clinical Medicine, LKS Faculty of Medicine, The University of Hong Kong, Hong Kong SAR, China; 2 The State Key Laboratory of Brain and Cognitive Sciences, The University of Hong Kong, Hong Kong SAR, China

**Keywords:** Perceived public stigma, Psychosis, Education level, Endorsement of medication treatment

## Abstract

**Objective:**

This study aimed to investigate the changes in perceived public stigma (PPS) towards psychosis, and endorsement of medication treatment between 2009 to 2018 in the Hong Kong Chinese population.The role of education level on the changes in PPS and endorsement of medication treatment for psychosis was also examined.

**Methods:**

Telephone survey of the general population was conducted in 2009, 2014, and 2018. PPS was assessed using the revised Link’s Perceived Discrimination-Devaluation Scale. Endorsement of medication was measured using an item asking if individuals with psychosis requires medication to manage their symptoms. Education level was separated into three categories (primary, secondary, and tertiary) for analysis. Factorial analysis of covariance was used to examine the main effects of survey year, education and endorsement of medication on stigma, and the interaction between survey year and education level, and survey year and endorsement of medication on PPS.

**Results:**

1016, 1018, and 1514 respondents completed the surveys in 2009, 2014, and 2018, respectively. PPS was found to be stable across the three public surveys. Endorsement of medication treatment was associated with higher PPS. An interaction effect between survey year and education level onPPS was observed. PPS was significantly lower and fewer respondents endorsed medication treatment in 2018 in the tertiary education group than in previous years.

**Conclusion:**

Current findings suggest that a targeted approach may be required for different education groups when developing anti-stigma public campaigns. Inclusion of other aspects of knowledge about psychosis may also be useful in reduction of PPS.

**Supplementary Information:**

The online version contains supplementary material available at 10.1186/s12888-022-04288-1.

## Background

Stigma towards psychosis, including perceived public stigma (PPS) significantly affects the recovery of individuals with the conditions [1, 2]. It is also higher than stigma towards other mental illnesses such as depression and anxiety disorders [3–7]. However, most large-scale anti-stigma public campaigns target mental illness in general, and few focus on psychosis specifically [8]. Within Asia, specifically in Japan and Hong Kong, a common approach to reduce stigma towards psychosis or schizophrenia was renaming the conditions to dispel the negative connotations associated with the original terms used [8–10]. Both regions observed effects of this approach on improving public stigma towards the conditions [9, 10].

Other anti-stigma programs for psychosis in Hong Kong were conducted using various approaches and coverage since the 2000s; these ranged from a public campaign targeting the general public to smaller-scale interventions targeting students and smaller groups [11–13]. These programs have included an educational element to improve knowledge and demythologize psychosis [11–13]. One of these programs was the Jockey Club Early Psychosis project (JCEP), launched in Hong Kong in 2009. It was a five-year project to deliver and evaluate a comprehensive intervention service to adults with first-episode psychosis between 26 to 55 years of age [11, 14]. One of its components is a five-year anti-stigma campaign to improve knowledge about psychosis and reduce stigma towards psychosis among the general public. The key knowledge included in the campaign was the nature and causes of psychotic illness and its possible treatments. Different platforms were used to disseminate this knowledge, including public talks, exhibitions, and mass media [14]. More than 100 talks and exhibitions to the general public were conducted during the campaign [11].

Medication has been suggested to be crucial in managing psychosis [15, 16]. While the findings of a study suggested that some individuals with psychosis may not require medication in the long-term [17], it was generally found that the risk of recurrence is greatly reduced early in the course of illness if patients adhered to their medication treatment [16]. Furthermore, it was found that the use of antipsychotic medications early in the course of illness was associated with relapse prevention, highlighting the importance of medication treatment [18]. It was also suggested that the use of medication treatment allowed for patients to remain in the community instead of requiring institutional care as medications helped with the management of the symptoms of these patients [15]. Hence, an endorsement of medication treatment in the public may reflect some degree of knowledge about the biological nature of the illness and its effective treatment.

Studies examining the long-term changes of endorsement of medication treatment and stigma about psychosis and their relationship are limited, with inconsistent findings. Most studies were from Western societies. A review conducted on studies covering a span of 11 years up till 2007 found that while there was a trend towards greater endorsement of medication treatment for psychosis, which paralleled greater acceptance of the biological explanation for psychosis through the decades, there was an increase in the desire for social distance, and the belief that individuals with psychosis were dangerous remained unchanged [19]. Similar findings were observed in a study conducted in Germany between 1993 and 2001 [20]. In the United States, it was found that while endorsement of formal treatment, including the use of medications and specialty care increased between 1996 and 2006, levels of public stigma remained stable during this period [21]. Even more recent data suggested that stigma towards schizophrenia, specifically, the perceptions that individuals with psychosis are violent has increased in 2018 in the United States [22]. A study in Austria examining attitudes towards psychosis over two decades (between 1998 and 2018) reported similar findings with those of Angermeyer and colleagues [20] with regards to greater endorsement of medication treatment and no change in ascription of violence to those with psychosis over time [23]. However, they found that there was an increase in acceptance towards those with psychosis in Austria over the two decades [23] compared to other studies that found an increase in desire for social distance [19, 20].

Education level was found to be related to stigma in various cross-sectional studies [24–30]. Findings from these studies were inconsistent. In Hong Kong, higher education level was associated with higher stigma towards psychosis amongst the general public [4, 24]. A recent study in Taiwan found higher education level was associated with lower prejudice and discrimination towards psychosis (including psychotic-like experience, attenuated psychosis syndrome, and schizophrenia) [31]. Furthermore, a study conducted in Hong Kong also observed differences in levels of public stigma towards attenuated psychosis syndrome and schizophrenia between respondents with different levels of education [32]. It has been suggested that those with higher education and socio-economic status may be more aware of and perceptive towards actual discriminatory attitudes, which may explain the relationship between higher education level and greater stigma [33]. Furthermore, people with higher education may be more aware of biological causal attributions and treatment for psychosis, which were found to be associated with more negative attitudes towards individuals with the conditions, especially regarding social distancing and avoidance [7]. Longitudinal studies conducted in various countries have observed a trend towards the biological explanation and endorsement of treatment for psychosis among the general public across the past two decades [21, 23], along with the increase in education levels of the public [34, 35]. Hence, it is possible that the relationship between higher education and stigma could be partly influenced by the increase knowledge about the biological nature of psychosis. However, few longitudinal studies investigated the effect of education level on changes in the endorsement of medication treatment, which may partially reflect endorsement of a biological explanation for psychosis [23], and stigma towards psychosis [19]. Results could have implications for establishing anti-stigma campaign strategies.

This study aimed to examine the changes in PPS, and endorsement of medication treatment for psychosis between 2009 to 2018 in the Hong Kong Chinese population,within the context of the various anti-stigma campaigns and programs conducted during the nine years. Findings will provide insight on the relationship between medication treatment endorsement, which partially reflects the endorsement of the biological nature of the illness, and PPS about psychosis over time in the general public. Factors associated with the change of PPS about endorsement of medication treatment for psychosis were also examined, focusing specifically on education level.

## Methods

### Study design and procedure

Three telephone surveys of the general population were conducted in Hong Kong over nine years (2009, 2014, and 2018) with similar methodology to ensure both comparability and compatibility. The three surveys were conducted between 25 March and 17 April 2009 [4], 14 March and 3 April 2014 [25] and 17 January and 26 January 2018 [24].

In each of the three surveys, telephone numbers were selected at random using telephone directories, and subsequent sets of phone numbers were generated using a "plus/minus one/two" method to ensure unlisted numbers were included. Duplicate phone numbers were removed, and the remaining numbers were randomized and included as the final sample. All three surveys were administered by experienced interviewers using the Web-based Computer-Assisted Telephone Interview (Web-CATI) system. Interviews were conducted between 18:30 and 22:30 on weekdays and other times of the day during weekends and public holidays. Each number was called up to five times before members of the household were considered non-contactable.

Only Cantonese-speaking Hong Kong Chinese residents who were 18 years old or above were included, and only one member from each target household was selected to complete the survey. The ‘last birthday’ rule, that is, interviewing the individual who had the most recent birthday on the day of contact, was applied in the 2009 and 2014 surveys. While the 'next birthday' rule, that is, interviewing the individual whose upcoming birthday was closest to the date of contact, was applied in the 2018 survey in households with more than one qualified member. Eligible respondents provided verbal informed consent before the administration of the structured surveys, which were conducted anonymously. Data collected by interviewers that did not meet a response rate of 40% of the overall contact list assigned were removed from the final sample to prevent non-response bias from these interviewers. Voice recording, screen capture, and on-site supervision were used to ensure the quality of the data, and audio clips of 7.4% of the final sample were checked by the research team and confirmed to be successful and completed (> 90% completion of the survey) [4].

Further details of the study methodology of the phone surveys for each year have been reported in previous studies [4, 24, 25]. The study protocols were approved by the Institutional Review Board of the University of Hong Kong/Hospital Authority Hong Kong West Cluster (Reference numbers: UW09-030 and UW17-540).

### Assessments

Socio-demographic details, including age, gender, and level of education, were collected. Respondents were split into three groups for further analysis based on their education level. These are (1) primary education group (≤ 6 years of education), (2) secondary education group (approximately 7 to 12 years of education, and (3) tertiary education group (≥ 13 years of education).

Perceived public stigma was assessed using the revised Chinese Link’s Perceived Discrimination-Devaluation Scale (LPDDS) [4, 36]. The 13 items in the revised LPDDS reflect different discriminatory and deprecatory attitudes towards individuals with psychosis. Respondents had to indicate how much they agree with the statements on the attitudes held by most people on a four-point Likert scale. Scores of all 13 items were summed up and averaged to obtain a total LPDDS score, with higher scores indicating higher levels of stigmatizing attitude towards people with psychosis. This scale was used because it has been suggested to be useful in addressing the issue of social desirability bias as respondents tended to provide favourable responses when asked about their own attitudes towards those with mental illness compared to the responses they expected from others [37, 38]. Hence, these questions on perceived attitudes of others would provide a more accurate picture of the PPS that individuals with mental illness are exposed to and hence an estimated level of public stigma towards mental illness [37]. The Cronbach’s alpha of the translated Chinese scale was 0.76.

A single-item question to assess endorsement of medication treatment for psychosis was used in all three surveys, asking whether people with psychosis required medication to control their symptoms. Response to this question was binary (yes/no).

### Statistical analyses

The sample of each of the three surveys was weighted for age, gender, and level of education using the rim-weighting method to account for differences between the sample and the general population of Hong Kong using the population census data closest to the survey year [4]. Descriptive statistics were conducted. Due to the large sample size, normality of data was assessed based on the skewness and kurtosis (-2 to 2 for both). The overall LPDDS scores of the entire sample across the three survey years were compared using the analysis of variance (ANOVA). Factorial analysis of covariance (ANCOVA) controlling for age, gender, and employment status was used to examine the main effects of survey year, education, and medication endorsement effects on PPS, as well as the interaction of survey year and education level, and survey year and medication endorsement on PPS. Further analyses were conducted to examine changes in LPDDS between the three survey years within each education group and medication endorsement group separately using ANOVA. Differences in LPDDS between the three education groups within each survey year were also assessed using ANOVA. Bonferroni post hoc test was used for pairwise comparisons. Chi-square test was conducted to compare the differences in endorsement of medication as treatment for psychosis between the three survey years in each education group separately to provide insights on its changes within each education group. All statistical analyses were conducted using SPSS version 25.0 (IBM Corp, Armonk, New York). Statistical significance was set a priori at *p* < 0.05 (two-tailed).

## Results

The number of completed interviews across the three surveys was 1016, 1018, and 1514 in 2009, 2014, and 2018, respectively. The effective response rates for the surveys, calculated using the international standard proposed by the American Association for Public Opinion Research [39], were 65.8% (2009), 66.7% (2014), and 56.2% (2018).

The mean age was 45.18 years (SD = 16.28), 45.84 years (SD = 17.04), and 48.98 years (SD = 18.15); and 46.4%, 45.4%, and 47.5% were males for the 2009, 2014 and 2018 surveys respectively. The gender breakdown was not significantly different across the three survey years. However, the respondents from the three survey years differed significantly in age, education attainment, and employment status (Table 1). The 2018 group had significantly older respondents, more respondents in the tertiary education group, and higher employment than the 2009 and 2014 survey groups (Table 1). Detailed demographic information of the study samples and the general population in Hong Kong based on the population census information closest to each survey time point are presented in Supplementary Table 1. The LPDDS score for all samples of the three surveys was normally distributed (Skewness = -0.091, -0.184, and -0.027; Kurtosis = 0.124, 0.028, and 0.489, for 2009, 2014, and 2018 respectively), and it was not significantly different between the three time points (*p* = 0.154) (Table 1).

**Table 1:** Demographic comparison between 2009, 2014, and 2018 groups after weighing (*n* = 3548)

	2009	2014	2018	Between-group differences	
	*n* = 1016	*n* = 1018	*n* = 1514	Test statistic	p
Gender (*n*, %)				χ^2^ = 1.11	0.574
Male	471 (46.4)	462 (45.4)	719 (47.5)		
Female	545 (53.6)	556 (54.6)	795 (52.5)		
Age, Mean (SD)	45.18 (16.28)	45.84 (17.04)	48.98 (18.15)	F = 42.81	< .01
Age group (*n*, %)*				χ^2^ = 44.35	< .01
18 – 29	197 (20.4)	180 (17.7)	248 (18.3)		
30 – 39	183 (18.9)	154 (15.1)	217 (16.0)		
40 – 49	206 (21.3)	155 (15.3)	233 (17.2)		
50 – 59	178 (18.4)	179 (17.6)	217 (16.0)		
60 and above	204 (21.1)	223 (34.3)	440 (32.5)		
Education (*n*, %)*				χ^2^ = 16.94	< .01
Primary or below	240 (23.6)	240 (23.6)	300 (19.8)		
Secondary	487 (48.1)	489 (48.1)	716 (47.3)		
Tertiary and above	286 (28.2)	287 (28.2)	496 (32.7)		
Employment status*				χ^2^ = 13.857	< .01
Employed	648 (64.0)	613 (60.5)	1010 (66.8)		
Unemployed	45 (4.4)	37 (25.2)	65 (4.3)		
Retired / Homemakers	319 (31.5)	363 (32.4)	437 (28.9)		
Q8^1^ (n, %)*				χ^2^ = 19.649	< .01
Yes	874 (86.2)	902 (89.1)	1211 (82.8)		
No	140 (13.8)	110 (10.9)	251 (17.2)		
LPDDS, Mean (SD)	2.63 (0.49)	2.67 (0.47)	2.65 (0.32)	F = 1.875	.154

^1^Q8, Question on whether patients with psychosis need prescription drugs to control their symptoms

^*^These variables contain missing data: 334 missing in age group, 7 missing in education level, 11 missing in employment status, and 60 missing in response on Q8

**Fig. 1:**
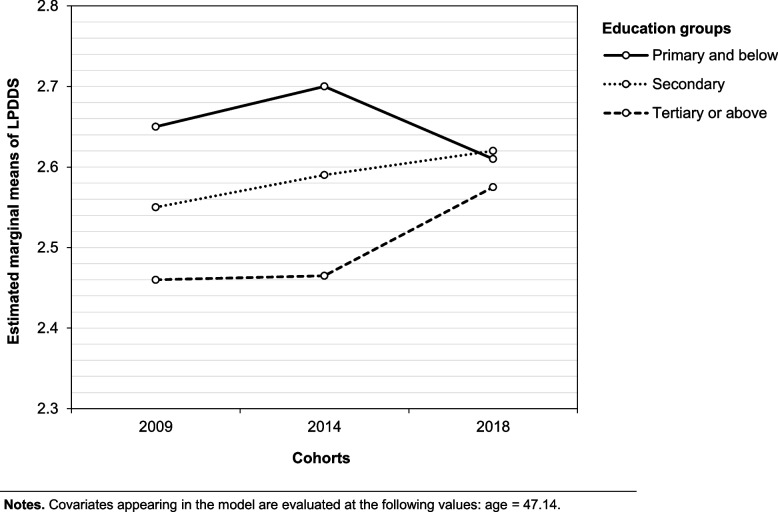
Estimated marginal means of Link’s Perceived Devaluation and Discrimination Scale between the three cohorts

Education level and endorsement of medication were positively associated with LPDDS score in the factorial ANCOVA analysis controlling for gender, age, and employment status (Table 2). While survey year did not have a main effect on PPS in the factorial ANCOVA analysis, an interaction effect was observed between survey year and education level (Table 2). Based on Fig. 1, the LPDDS scores for those in the secondary education group remained stable across the three survey years. The respondents within the tertiary education group had lower LPDDS in 2018 than those in 2014, and the opposite was observed in the primary education group (Fig. 1). Further analysis revealed that the lower LPDDS score in the 2018 group compared to previous years observed in Fig. 1 for the tertiary education group was statistically significant (Table 3).

**Table 2:** Effects of survey year, education and endorsement of medication treatment on perceived public stigma towards psychosis

Predictor	Sum of Squares	*df*	Mean Square	*F*	*P*	Partial η^2^
Survey year	0.608	2	0.304	1.754	.173	0.001
Gender	0.462	1	0.462	2.670	.102	0.001
Age	0.235	1	0.235	1.359	.244	0.000
Employment group	0.053	2	0.026	0.153	.859	0.000
Education group	5.799	2	2.900	16.745**	< .001	.011
Q8^1^	8.257	1	8.257	47.683**	< .001	0.015
Survey year x Education group interaction	3.081	4	0.770	4.448**	.001	0.006
Survey year x Q8 interaction	0.767	2	0.383	2.214	.109	0.001

^1^Q8, Question on whether patients with psychosis need prescription drugs to control their symptoms

**Table 3:** Perceived public stigma score (LPDDS) of education group and endorsement of medication treatment across three survey years

	2009	2014	2018	Between-group differences	
				Test statistic	*p*
Education group					
Primary	2.52 (0.42)	2.53 (0.47)	2.60 (0.37)	F = 2.751	.065
Secondary	2.63 (0.50)	2.65 (0.46)	2.66 (0.32)	F = 0.958	.384
Tertiary	2.73 (0.49)	2.80 (0.45)	2.65 (0.28)	F = 14.108**	< .001
Q8^1^					
Yes	2.66 (0.48)	2.69 (0.47)	2.66 (0.32)	F = 1.761	.172
No	2.49 (0.51)	2.52 (0.49)	2.60 (0.33)	F = 3.150*	.044

^1^Q8, Question on whether patients with psychosis need prescription drugs to control their symptoms

Furthermore, the three education groups differed significantly on their LPDDS scores in 2009 and 2014 (Supplementary Table 2. The tertiary education group reported the highest PPS score, and the primary education group had the lowest PPS score (Supplementary Table 2). However, in 2018, while the secondary education group had significantly higher PPS score than the primary education group, the PPS level of the tertiary education group was not significantly different from the other two education groups (Supplementary Table 2).

Regarding medication treatment endorsement, significantly fewer respondents agreed that individuals with psychosis required medication treatment in 2018 than in the two previous survey years (Table 1). Significantly more respondents in the primary education group endorsed the use of medication for managing psychosis in 2014 and 2018 compared to the 2009 survey (Table 4). In the tertiary education group, significantly fewer respondents endorsed medication treatment for psychosis in 2018 than previous survey years (Table 4). No significant difference was observed between the three survey years in the secondary education group (Table 4).

**Table 4:** Comparison of responses on endorsement of medication treatment of each education group across survey

	2009	2014	2018	Between-group differences	
Primary and below (*n*, %)				Test statistic	*p*
				χ^2^ = 9.383**	.009
Q8^1^ Yes	208^a^ (86.7)	224^b^ (93.7)	269^b^ (93.1)		
Q8^1^ No	32^a^ (13.3)	15^b^ (6.3)	20^b^ (6.9)		
Secondary (*n*, %)				χ^2^ = 3.988	.136
Q8^1^ Yes	418^c^ (86.0)	422^c^ (86.7)	568^c^ (82.8)		
Q8^1^ No	68^c^ (14.0)	65^c^ (13.3)	118^c^ (17.2)		
Tertiary and above (*n*, %)				χ^2^ = 22.688**	< .001
Q8^1^ Yes	245^d^ (86.0)	254^d^ (89.4)	372^e^ (76.9)		
Q8^1^ No	40^d^ (14.0)	30^d^ (10.6)	112^e^ (23.1)		

The same subscript letters across survey years in each education group represents a subset of survey year categories where column proportions do not differ significantly from each other at .05 level

^1^Q8, Question on whether patients with psychosis need prescription drugs to control their symptoms

## Discussion

The level of PPS was stable across the three public surveys conducted in 2009, 2014, and 2018 in Hong Kong (2.63, 2.67, and 2.65, respectively). These scores were comparable with those observed from an earlier study conducted amongst the Belgian general public in their attitudes towards individuals with schizophrenia or major depression [40]. PPS was significantly associated with education level and endorsement of medication treatment for psychosis across the three survey years. Endorsement of medication treatment was associated with higher PPS. An interaction effect between survey year and education level on PPS levels was observed. Further analyses found that PPS was significantly lower in 2018 for the tertiary education group than in previous years. In 2009 and 2014, the tertiary education group had the highest stigma, and the primary education group had the lowest level of PPS. In 2018, the PPS score of the tertiary education group was not significantly different from the other two education groups. Furthermore, significantly fewer respondents in the tertiary education group endorsed the use of medication in 2018 compared to 2009 and 2014. Significant increase in endorsement of medication treatment over the nine years was observed among the primary education group, while no significant change was seen among the secondary education group over the period.

The findings from the current study suggested that PPS about psychosis was relatively stable at the population level in Hong Kong, even with a range of anti-stigma public campaigns conducted over the period [11–13]. This was inconsistent with an earlier study conducted in Germany across 21 years that observed a significant reduction in PPS towards those with mental illness from 1990 to 2011, particularly amongst the younger population [41]. However, the study conducted in Germany examined PPS towards individuals with mental illness in general, which may differ from PPS towards psychosis, and that has been observed to be more pronounced [5–7]. A study assessing the effects of an anti-stigma training (focusing mainly on psychoeducation) in China found that participants in the intervention group had significantly lower scores in PPS towards those with serious mental illness compared to their counterparts in the control group after the intervention was administered [42]. However, this study focused on a specific population – primary healthcare workers who provide assistance to mental healthcare professionals, which may not represent the general public. Findings from the results of the current study suggest that within Hong Kong, while campaigns at the population level may be useful for managing stigma towards mental illnesses in general, tailored interventions for specific populations may be required to reduce stigma towards severe mental illness such as psychosis. Further studies on the long-term effects of these interventions are needed.

Our findings were partially consistent with that of earlier studies. Similar to earlier studies, we found that there was an increase in endorsement of medication treatment for psychosis between 2009 and 2014 [19–21, 23], however, unlike earlier studies, we observed a decrease in medication endorsement in 2018. These changes coincided with the timeline of the public campaign period of JCEP, suggesting that the campaign had an effect on advocating the use of medication treatment for psychosis, a possible reflection of the biological nature of the illness, amongst the general population. There was a lack of change in overall PPS level despite the changes in endorsement of medication treatment observed across the three survey years at population level. It has been suggested that targeting commonly adopted stereotypes of individuals with psychosis, and using a bio-psycho-social framework for education, may be more useful in reducing PPS towards psychosis [43]. Apart from didactic knowledge, using contact-based video in anti-stigma interventions was also found to result in a greater reduction of stigmatizing attitudes towards schizophrenia in students and adults [12, 13, 44] and could be adopted in future public campaigns.

The current study found different directions in the relationship between education level and PPS in different survey years. Higher PPS levels were found among individuals with higher education levels in 2009 and 2014 but not in 2018. This suggests that the nature of the relationship between education level and PPS of psychosis is unstable and likely to change with different circumstances. This study further examined the changes in PPS and endorsement of medication treatment for psychosis of each education group in detail over the years. We found that individuals within the secondary education group had stable levels of PPS and endorsement of medication treatment across the three survey years. However, a gradual increase in PPS was observed in those with lower education levels over the years along with an increase in their endorsement of medication treatment for psychosis. On the other hand, for the tertiary education group, we found stable levels of PPS between 2009 to 2014 and a significant reduction in 2018. At the same time, significantly fewer respondents in the tertiary education group of the 2018 survey endorsed medication treatment for psychosis compared with the previous years. These patterns provided convergent evidence that endorsement of medication treatment, which may be associated with a trend towards acceptance of a biological model for explaining psychosis [23] is associated with higher level of PPS. Furthermore, the PPS and endorsement of medication treatment for psychosis among individuals in primary and tertiary education groups may be more malleable and responsive to external influences, including public campaigns. In contrast, those of the secondary education group may be more static. The different directions of changes in medication endorsement and stigma among primary education and tertiary education groups between 2014 and 2018 suggest that the campaign's effect may be more persistent within these groups. These findings highlight that public anti-stigma campaigns may require different approaches for different education groups.

### Limitations

This study had several limitations. Selection bias may be an issue because only fixed-line telephone numbers were used in this study and the relatively low response rates in the three surveys. This also limited the generalizability of the study results. This study only assessed endorsement of medication treatment for managing symptoms of psychosis; other factors that are widely reported to be commonly associated with stigma towards mental illness, for instance, exposure to and contact with individuals with psychosis were not assessed in this study. Inclusion of measures assessing behavioural discrimination would also have strengthened the study findings by providing insights on other aspects of stigma. Finally, there was a lack of measures assessing respondents' exposure to the JCEP anti-stigma campaign and other anti-stigma programs conducted in Hong Kong during the study time period. Hence, definitive conclusions could not be drawn on whether the changes observed in the study were directly due to the anti-stigma programs conducted during the time assessed in the study.

## Conclusion

The current study found that perceived public stigma is static among the general public in Hong Kong over nine years despite an increase in endorsement of medication treatment for psychosis. Different education groups had different patterns of changes in PPS and endorsement of medication treatment across the years. These suggest that different population groups require tailored approaches to the anti-stigma public campaigns to reduce PPS. Finally, further investigations are required to explore the sustainability of public anti-stigma campaigns.

## Supplementary Information

**Additional file 1: Supplementary Table 1.** Demographic characteristics of study samples and the total population.

**Additional file 2: Supplementary Table 2.** Public stigma score (LPDDS) differences between the three education groups in each survey year.

## Data Availability

The datasets used and analysed during the current study are available from the corresponding author on reasonable request.

## Abbreviations

JCEP: Jockey Club Early Psychosis project
Web-CATI: Web-based Computer-Assisted Telephone Interview
LPDDS: Link’s Perceived Discrimination-Devaluation Scale
ANOVA: Analysis of Variance
ANCOVA: Analysis of Covariance
SPSS: Statistical Package for Social Sciences
SD: Standard Deviation
DF: Degrees of Freedom
Partial η2: Partial Eta-squared
PPS: Perceived public stigma
UK: United Kingdom
