# Supplementary material for: The role of education level on changes in endorsement of medication treatment and perceived public stigma towards psychosis in Hong Kong: comparison of three population-based surveys between 2009 and 2018
Source: BMC Psychiatry. 2022 Oct 13;22:641. doi: 10.1186/s12888-022-04288-1 (PMC9559020; doi:10.1186/s12888-022-04288-1)
Supplement: Supplementary file 1 — Additional file 1: Supplementary Table 1. Demographic characteristics of study samples and the total population. [file 12888_2022_4288_MOESM1_ESM.docx]

Supplementary Table 1. Demographic characteristics of study samples and the total population

|  | 2009 | | 2014 | | | 2018 | | |
| --- | --- | --- | --- | --- | --- | --- | --- | --- |
|  | Survey  (n = 1016), % | Total population^a^, % | | Survey  (n = 1018), % | Total population^b^, % | | Survey  (n = 1514), % | Total population^c^, % |
| Gender |  |  | |  |  | |  |  |
| Male | 46.4 | 46.6 | | 45.4 | 45.6 | | 47.5 | 47.5 |
| Female | 53.6 | 53.9 | | 54.6 | 54.4 | | 52.5 | 52.5 |
| Age group |  |  | |  |  | |  |  |
| 18 – 29 | 20.4 | 23.5 | | 17.7 | 21.3 | | 18.3 | 17.6 |
| 30 – 39 | 18.9 | 18.4 | | 15.1 | 17.7 | | 16.0 | 16.4 |
| 40 – 49 | 21.3 | 21.3 | | 15.3 | 18.3 | | 17.2 | 17.7 |
| 50 – 59 | 18.4 | 17.2 | | 17.6 | 19.3 | | 16.0 | 20.7 |
| 60 and above | 21.1 | 19.6 | | 34.3 | 23.4 | | 32.5 | 27.6 |
| Education |  |  | |  |  | |  |  |
| Primary or below | 23.6 | 29.3 | | 23.6 | 29.3 | | 19.8 | 20.6 |
| Secondary | 48.1 | 46.6 | | 48.1 | 46.6 | | 47.3 | 46.2 |
| Tertiary and above | 28.2 | 24.1 | | 28.2 | 24.1 | | 32.7 | 33.2 |

^a^Gender and age group were based on mid-2008 census data, education group was based on 2011 census data
^b^Gender and age group were based on mid-2013 census data, education group was based on 2011 census data
^c^Gender, age group and education group were based in 2016 census data
