# Supplementary material for: The role of education level on changes in endorsement of medication treatment and perceived public stigma towards psychosis in Hong Kong: comparison of three population-based surveys between 2009 and 2018
Source: BMC Psychiatry. 2022 Oct 13;22:641. doi: 10.1186/s12888-022-04288-1 (PMC9559020; doi:10.1186/s12888-022-04288-1)
Supplement: Supplementary file 2 — Additional file 2: Supplementary Table 2. Public stigma score (LPDDS) differences between the three education groups in each survey year. [file 12888_2022_4288_MOESM2_ESM.docx]

Supplementary Table 2. Public stigma score (LPDDS) differences between the three education groups in each survey year

|  | Primary | Secondary | Tertiary | Between-group differences | |
| --- | --- | --- | --- | --- | --- |
|  |  |  |  | Test statistic | p |
| Survey year |  |  |  |  |  |
| 2009 | 2.52 (0.42)^a^ | 2.63 (0.50)^b^ | 2.73 (0.49)^c^ | F = 11.786** | <.001 |
| 2014 | 2.53 (0.47)^d^ | 2.65 (0.46)^e^ | 2.80 (0.45)^f^ | F = 21.932** | <.001 |
| 2018 | 2.60 (0.37)^g^ | 2.66 (0.32)^h^ | 2.65 (0.28)^g,h^ | F = 4.441* | .012 |

The same subscript letters across the education groups in each survey year represents a subset of education group categories where column proportions do not differ significantly from each other in the post hoc Bonferroni test.
